# Beneficial Effects of Polyphenol-Rich Food Oils in Cardiovascular Health and Disease

**DOI:** 10.31083/j.rcm2407190

**Published:** 2023-07-03

**Authors:** Lucia Kindernay, Kristína Ferenczyová, Veronika Farkašová, Ulrika Duľová, Jakub Strapec, Monika Barteková

**Affiliations:** ^1^Institute for Heart Research, Centre of Experimental Medicine, Slovak Academy of Sciences, 84104 Bratislava, Slovakia; ^2^Institute of Physiology, Faculty of Medicine, Comenius University in Bratislava, 81372 Bratislava, Slovakia

**Keywords:** food oils, polyphenols, cardiovascular disease, hypertension, atherosclerosis, heart hypertrophy, cardioprotection

## Abstract

A variety of vegetable and fruit derived food oils are considered beneficial for human health due to their content of functional components including their positive effects in cardiovascular system. In addition to the favorable ratio of unsaturated versus saturated fatty acids, some of these oils include also other health beneficial compounds such as vitamins, minerals, pigments, enzymes and phenolic compounds. Particularly polyphenols have been documented to exert numerous positive effects in cardiovascular system including their anti-hypertensive, anti-atherogenic as well as cardio- and vasculo- protective effects in subjects suffering from various cardiovascular and cardiometabolic diseases, likely via their antioxidant, anti-inflammatory, anti-coagulant, anti-proliferative and anti-diabetic properties. However, it has not been proven so far whether the positive cardiovascular effects of polyphenol-rich food oils are, and to what measure, attributed to their phenolic content. Thus, the current review aims to summarize the main cardiovascular effects of major polyphenol-rich food oils including olive, flaxseed, soybean, sesame and coconut oils, and to uncover the role of their phenolic compounds in these effects.

## 1. Introduction

Nutritional factors play a dominant role in maintaining cardiovascular health in both positive and negative ways: while healthy food and an appropriate diet help to keep healthy heart and vessels, unhealthy food and inappropriate diet (especially overeating leading to overweight and obesity) significantly contribute to the development of cardiovascular and cardiometabolic (metabolic disorders that contribute to increased cardiovascular morbidity and mortality) diseases. Moreover, various healthy so called “functional foods” contain certain health beneficial compounds, e.g., vitamins, minerals, pigments, enzymes, phenolic compounds or polyunsaturated fatty acids (PUFA) that may particularly efficiently contribute to the prevention of cardiovascular and cardiometabolic diseases via their antioxidant, anti-inflammatory, anti-coagulant, anti-proliferative or anti-diabetic properties [1, 2, 3]. Among foods that are considered potentially beneficial for cardiovascular health belong vegetable and fruit derived food oils used either for consumption in the fresh form or for cooking, particularly those containing a beneficial ratio of saturated versus unsaturated fatty acids (FA), especially those with high content of PUFA. However, many widely or rarely used food oils contain also other (non-lipid) potentially beneficial components that may contribute to their positive effects on the cardiovascular system, among whose polyphenolic substances with strong antioxidant potential play a pivotal role. Since polyphenols as well as other plant-derived natural antioxidants have been widely documented to exert positive effects on cardiovascular and cardiometabolic health [4, 5, 6], it may implicate that beneficial effects of polyphenol-enriched food oils in cardiovascular system may be, at least in part, attributed to their phenolic content. In line with this view, the the current review aims to summarize the effects of major polyphenol-rich food oils, namely olive oil, flaxseed oil, soybean oil, sesame oil, coconut oil and some others on the most frequent cardiovascular pathologies including hypertension, heart hypertrophy, atherosclerosis and ischemic heart disease. The inclusion criteria for the food oils to be mentioned in the paper were (1) significant content of the polyphenol component in the particular oil; and (2) common usage of the particular oil for the cooking and meal preparation (excluded oils used in cosmetics or pharmacy). In addition, an important aim of the current paper was to uncover whether polyphenolic components of food oils may play a significant role in their cardiovascular effects.

## 2. The Effect of Olive Oil on Cardiovascular Health and Diseases

Olive oil (OO), concretely extra virgin OO consists mostly of monounsaturated FA (ranging 65.2–80.8%), polyunsaturated FA (ranging 7.0–15.5%), other lipids, tocopherols, carbohydrates, pigments [7] and other compounds such as phenols and sterols [8]. Despite the high content of monounsaturated FA in OO, which has been widely associated with its health-promoting properties [9], OO contains a wide spectrum of bioactive substances (2% of total weight), that differ between olive fruits and the different olive oils [10, 11]. Polyphenols have been widely acknowledged as the most relevant of these bioactive compounds. The most abundant polyphenols in OO — oleuropein, hydroxytyrosol and tyrosol are outstanding for their bioactive features [12]. In particular, these OO compounds have shown cardioprotective potential due to their anti-oxidant and anti-inflammatory properties [13, 14].

### 2.1 Olive Oil and Olive Oil Polyphenols Treatment/Supplementation Impact on Cardiac Injury

Myocardial damage due to ischemia or myocardial infarction (MI) causes an increase in oxidative stress, pro-inflammatory response and the production of different cytokines. Under chronic conditions, these changes contribute to cardiovascular remodeling and heart failure [15].

#### 2.1.1 Evidence from Clinical Studies

A recent PREDIMED study on 7447 participants concluded that in the group supplemented with extra virgin OO (≥4 tablespoon/day, median follow-up of 4.8 years) in Mediterranean diet (MedDiet) reduces by 30% the risk of major cardiovascular events as MI and stroke compared to the control group [16]. Results of the same study also showed significantly reduced risk of atrial fibrillation in the group with extra virgin OO food supplementation [17]. A case-control study based on validated semi-quantitative food frequency questionnaire demonstrated that OO food intake (median intake: 54 g/day) was associated with a statistically significant 82% relative reduction in the risk of a first MI [18]. In another study where the diet was assessed using food frequency questionnaires, the higher OO intake (>0.5 tablespoon/day or >7 g/day, during 24 years) was associated with a 14% lower risk of cardiovascular disease (CVD) and 18% lower risk of coronary heart disease [19]. Moreover, daily moderate consumption of virgin OO (1 and 1/2 tablespoons, mean follow-up of 10.7 years), compared to common OO, was associated with half the risk of cardiovascular mortality in Mediterranean population [20].

#### 2.1.2 Evidence from Animal Studies

**Fig. 1.**
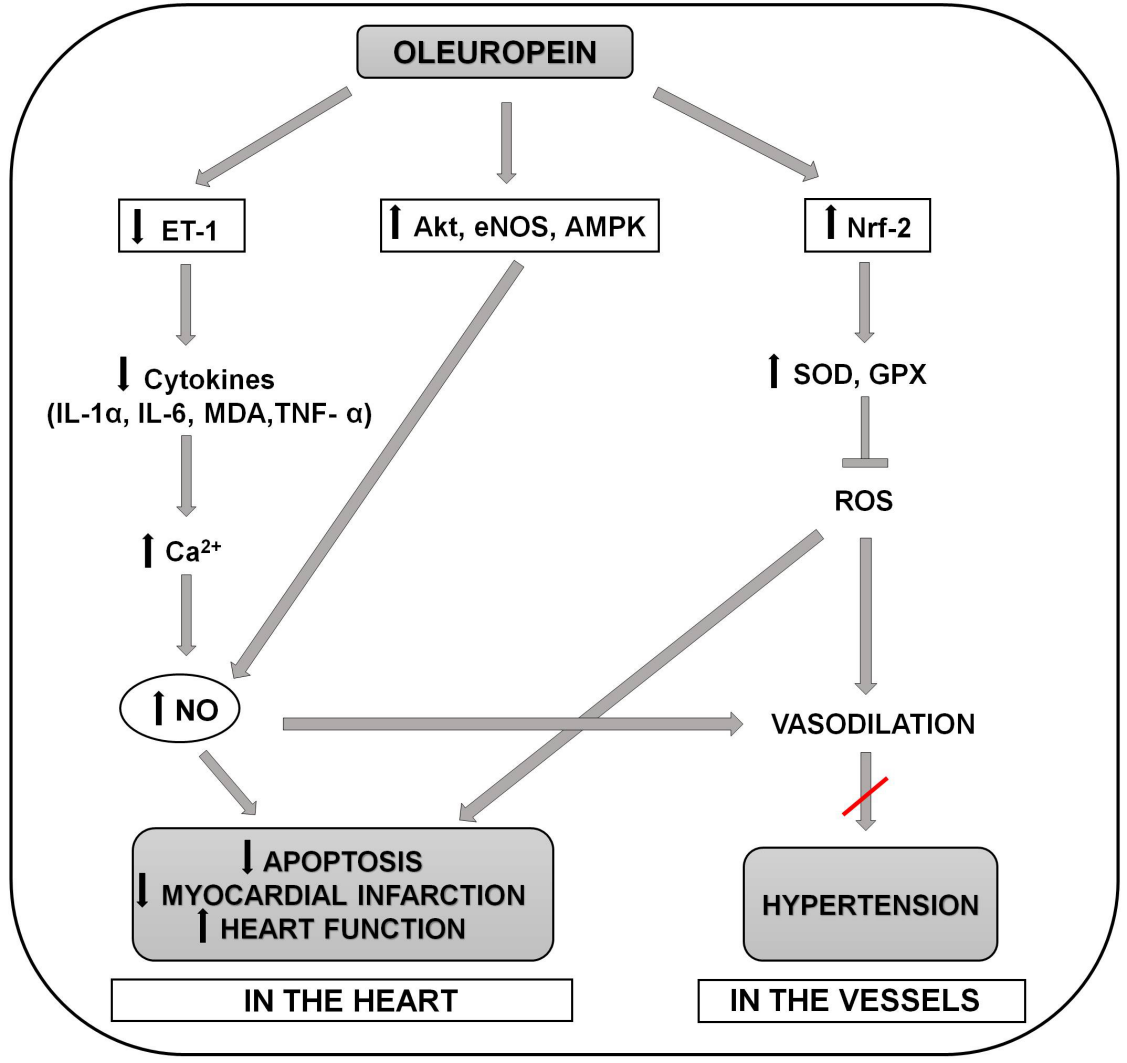
**Schematic representation of the molecular mechanisms involved in the positive effects of oleuropein in preventing the development of hypertension and myocardial infarction and apoptosis in the heart**. Oleuropein reduces release of proinflammatory cytokines (serum MDA, IL-6,IL-1α and TNF-α) and increases intracellular calcium, through inactivation of endothelin-1. Increase in intracellular calcium then cause increase in NO production, which leads to decrease of apoptosis, reduction of myocardial infarct size and improvement of the heart function. Oleuropein also could increase NO production by activation of AMPK/Akt/eNOS signaling pathways. Moreover, increase in NO production leads to vasodilation and ameliorate the development of hypertension. Oleuropein also provides antioxidant defense (blocking ROS production) trough the activation of Nrf-2 and increase in SOD and GPX levels. This process leads not only to vasodilation and anti-hypertensive effect, but also to decrease of apoptosis, reduction of myocardial infarct size and improvement of the heart function. Akt, protein kinase B, AMPK, 5’ adenosine monophosphate-activated protein kinase; Ca, calcium; ET-1, endothelin 1; eNOS, endothelial nitric oxide synthase; GPX, glutathione peroxidase; IL-1α, interleukin 1 alpha; IL-6, interleukin 6; MDA, malondialdehyde; NO, nitric oxide; Nrf-2, nuclear factor erythroid 2–related factor 2; ROS, reactive oxygene spieces; SOD, superoxide dismutase; TNF-α, tumor necrosis factor-α.

In an experimental study in rats, a standard diet supplemented with extra virgin OO (10% w/w, 10 days before left anterior descending (LAD) artery ligation + 16 weeks post-LAD ligation) protected against left ventricular dysfunction throughout improved left ventricular ejection fraction and prevented from adverse cardiac remodeling post MI. Moreover, extra virgin OO was able to decrease tumor necrosis factor-α (TNF-α) level and oxidative stress in heart after MI [21]. Similarly, rats fed with a diet enriched with extra virgin OO (16 weeks) exhibited significantly improved systolic and diastolic post-ischemia/reperfusion (I/R) function and reduced the size of MI [22]. Moreover, a wide range of animal studies found oleuropein and hydroxytyrosol food supplementation to offer cardioprotection against MI in a model of acute MI and heart failure [23, 24, 25, 26] or even in models of drug-induced cardiomyopathies [27, 28, 29]. These effects attributed to oleuropein are provided by increased antioxidant levels (superoxide dismutase, glutathione reductase), by inducing antioxidant defense genes through nuclear factor erythroid 2–related factor 2 (Nrf-2) axis [26], by the reduced release of proinflammatory cytokines (serum malondialdehyde, interleukin-1β, and TNF-α) [23, 25] and by attenuation of the reperfusion-induced calcium overload [24]. In models of drug-induced cardiomyopathies, the main effect of oleuropein seems to be the increase of Akt, AMP-activated protein kinase (AMPK), and endothelial nitric oxide synthase (eNOS) phosphorylation (restored nitric oxide (NO) bioavailability), independently of the presence of oxidative stress [27, 28] (Fig. 1).

### 2.2 Hypertension and Olive Oil Supplementation

Hypertension is one of the major risk factors for developing CVD, the biggest single contributor to the global burden of disease and mortality. There is evidence that the pattern of the MedDiet rich in OO in addition to other elements may offer a considerable benefit against the risk of hypertension, type 2 diabetes mellitus and CVD [16].

#### 2.2.1 Evidence from Clinical Studies

A clinical trial by Sarapis* et al*. [30] observed a significant decrease in peripheral and central systolic blood pressure (BP) after 3-week high polyphenol OO (60 mL/day) consumption in healthy participants. Also, prolonged 8-week consumption of diets containing polyphenol-rich OO (∼30 mg/day) has been associated with decreased BP and improvement in endothelial function in women with high-normal or stage 1 essential hypertension [31]. Similarly, in women with excess body fat, the ingestion of 25 mL of extra virgin OO daily for 8 weeks has been shown to reduce systolic and diastolic BP while improving body fat [32]. Furthermore, MedDiet supplemented with extra virgin OO (52 g/day for 1 year) decreased BP in hypertensive women [33]. Moreover, Hohmann* et al*. [34] in a meta-analysis demonstrated that ingestion of high-polyphenolic extra virgin OO was associated with systolic BP reduction. Similarly, data from the Brisighella Heart Study showed prevalent consumption of extra virgin OO as a main seasoning and cooking fat source was significantly associated to lower blood pressure and arterial stiffness, when compared with predominantly animal fat users [35]. These anti-hypertensive effects of extra virgin OO can be attributed to their polyphenols which stimulate NO production and inhibit the expression of endothelin-1 which plays a crucial role in reducing BP [33]. On the other hand, several studies documented negative results of OO use for BP reduction. For example, no significant effects on systolic or diastolic BP were observed after a single dose of high polyphenolic extra virgin OO in patients with prediabetes and metabolic syndrome (50 mL or 35 g) [36]. Even 4-week consumption of extra virgin OO in a dose of 50 g/day had no significant effect on systolic and diastolic BP despite its beneficial effects on circulating lipid profiles in healthy individuals [37].

#### 2.2.2 Evidence from Animal Studies

Clinical data on the anti-hypertensive effects of extra virgin OO are also supported by a range of animal models. Recent studies using extra virgin OO (20%) or wild OO (15%) dietary supplementation for 12 weeks demonstrated a reduction in both systolic and diastolic BP in hypertensive rats [38, 39]. This hypotensive effect of OO might be exerted, at least in part, in an endothelium-dependent manner via improving NO bioavailability. Moreover, an OO-enriched diet alleviates vascular dysfunction, improves vascular remodeling and reduces aortic fibrosis in hypertensive rats [39]. Improvement of endothelial function after administration of OO can be attributed to the inhibition and/or scavenging of reactive oxygen species (ROS) [40]. This may suggest a decrease in oxidative stress, probably associated with the effect of polyphenolic components of extra virgin OO with antioxidant properties such as hydroxytyrosol and/or oleouropein [41]. Subsequently, a decrease in systolic BP was also observed in spontaneously hypertensive rats (SHR) after enriching a virgin OO with phenolic compounds [42] indicating an association between olive polyphenols and positive BP outcomes [43]. The antihypertensive effects of one of the most abundant OO polyphenols- oleuropein, might be partly mediated by improving the release of nitric oxide and antioxidant and sympatholytic activities [44]. In addition, high-polyphenolic extra virgin OO has been found to contain peptides and water-soluble extracts that inhibit angiotensin-converting enzyme which has anti-hypertensive effects in SHR [45]. On the other hand, Terés* et al*. [46] demonstrated that the hypotensive effect of OO is caused by its high oleic acid content.

### 2.3 Atherosclerosis and Olive Oil Supplementation

Bioactive compounds of OO have the potential to reduce oxidative stress and improve endothelial function through their anti-oxidant, anti-inflammatory, and anti-thrombotic properties, therefore reducing the risk and progression of atherosclerosis [47].

#### 2.3.1 Evidence from Clinical Studies

The recent PREDIMED clinical trial (Prevención con Dieta Mediterránea) and other studies that compared major CVD in participants receiving diets supplemented with extra virgin OO (≥4 tablespoon/day, median follow-up of 4.8 years) provide evidence to support the atheroprotective value of OO in the context of MedDiet [16]. Long-term consumption of a MedDiet rich in extra virgin OO (≥4 tablespoons/day; 10–15 g/tablespoon; follow-up period of 5 and 7 years) was associated with decreased atherosclerosis progression, as shown by reduced intima-media thickness of both common carotid arteries and carotid plaque height [48]. The results of another study in elderly persons at high cardiovascular risk suggest that long-term adherence to a MeDiet plus extra virgin OO (dosage not specified, 3- and 5-year follow-up) could delay atheroma plaque development by reducing rolling, adhesion, and migration processes of circulatory mononuclear cells into the arterial wall. In addition, this data suggest that the MeDiet plus extra virgin OO also decreases plaque vulnerability by lowering instability factors interleukins (IL-18) and increasing stability factors (IL-10 and IL-13) [49]. Moreover, the meta-analysis showed that OO supplementation is associated with a reduction in CVD incidence including acute coronary syndrome, stroke, and peripheral arterial disease in both high-risk and healthy patients [50].

Even acutely administrated high polyphenolic extra virgin OO (50 mL or 35 g single dose) improved endothelial function measured as flow-mediated vasodilation as compared to refined OO [36] or butter [51] in patients with type 1 diabetes mellites [51] or prediabetes [36]. Additionally, consumption of extra virgin OO is associated with a reduction in inflammatory biomarkers and molecules implicated in atherosclerosis as well as CVD incidence and mortality and these anti-inflammatory and cardioprotective effects of extra virgin OO are mostly attributable to its high content of polyphenol molecules [52]. Interesting results brought also studies comparing anti-atherogenic potential of virgin OO enriched with additional phenolic content (either with its own polyphenols or with phenolic substances from other sources). In this regards, a randomized, double-blind, crossover, controlled trial in 33 hypercholesterolemic individuals receiving 25 mL/day of standard virgin OO or virgin OO enriched with its polyphenols or those of thyme documented that polyphenols from olive oil and thyme modified the plasma lipoprotein profile and decreased the atherogenic ratios: low-density lipoprotein (LDL)/high-density lipoprotein (HDL) particles, small HDL/large HDL, and HDL-cholesterol (HDL-C)/HDL-Particle Number (HDL-P), and decreased the lipoprotein insulin resistance index. The results indicate that OO polyphenols, and those from thyme, provide benefits on lipoprotein particle atherogenic ratios and subclasses profile distribution [53]. In addition, both polyphenol-enriched olive oils (with own polyphenols and with those of thyme) increased HDL antioxidant content, and thyme polyphenol-enriched OO also increased α-tocopherol, the main HDL antioxidant, in hypercholesterolemic subjects [54]. Thus, enrichement of OO with phenolic compounds might be a way to increase the healthy properties of OO including its anti-atherogenic properties.

#### 2.3.2 Evidence from Animal Studies

It has been shown that extra virgin OO-enriched diet for 8 weeks ([55]; 5 mL/kg/day) or 12 weeks ([56]; dosage not specified) was associated with a reduction in the early development of atherosclerosis and fatty streak formation in the aorta and coronary arteries of rabbits and rats on hypercholesterolemic diet [55, 56]. Similarly, the diet supplemented with virgin OO (1.75 g virgin OO/100 g standard chow for 30 days) stops the progression of aortic atherosclerotic lesions in rabbits on a hypercholesterolemic diet [57]. Paknahad* et al*. [58] also showed a significantly lower degree of aortic atheromatous lesions in hypercholesterolemic rabbits supplemented with OO (8% w/w) for 12 weeks. Furthermore, Lian* et al*. [59] demonstrated lowered atherosclerotic lesion area of the whole aorta and aortic sinus in Ldlr–/– mice (a mouse model of atherosclerosis) after 3 and 6 months on the diet supplemented with extra virgin OO and nuts. This diet also reduced monocyte expression of inflammatory cytokines, CD36, and CD11c, with decreased monocyte uptake of oxidized LDL *ex vivo* and reduced CD11c+ foamy monocyte firm arrest on vascular cell adhesion molecule-1 [59]. Interesingly, Luque-Sierra* et al*. [60] demonstrated that extra virgin OO with a higher content of phenolic compounds does not provide further benefits in the prevention of atherosclerosis in comparison to extra virgin OO with a natural content of phenolic compounds in Ldlr-/-.Leiden Mice. On the other hand, enrichment of extra virgin OO with green tea polyphenols further improved beneficial antiatherogenic effects of natural extra virgin OO in the atherosclerotic apolipoprotein-E-deficient mice [61].

## 3. The Effect of Flaxseed Oil on Cardiovascular Health and Diseases

Flaxseed oil, also known as linseed oil, is bright yellow oil obtained by cold pressing (to maintain the antioxidants and prevent them from heat damage) from dried ripened seeds of the flax plant. Flaxseed oil is known for a variety of its health benefits and practical uses (i.e., in the kitchen, for skin care or as a nutritional supplement). In general, flaxseed is one of the richest plant sources of FA with the most represented essential ω-3 (n-3) FA - α-linolenic acid (n-3 ALA) in the range of 39.9–60.5%. The FA profile of flaxseed oil is further composed of ω-6 linoleic (12.3–17.5%), oleic (13.4–19.4%), stearic (2.2–4.6%), and palmitic (4.9–8%) acids. Flaxseed oil has the potential to exert several cardio and vasculoprotective effects due to it involves a precursor for endogenous synthesis of docosapentaenoic acid (DHA) and eicosapentaenoic acid (EPA) [62] and due to it contains an important lignan - secoisolariciresinol diglucoside (SDG), a potent antioxidant, together with some other lignans in much smaller concentrations, namely matairesinol, lariciresinol and pinoresinol [63]. In dark bottles, flaxseed oil is stable at refrigeration temperatures for up to 6 months, whereas at room temperature it is sensitive and can be spontaneously oxidized within one week [64].

### 3.1 Effects of Flaxseed Oil in Hypertension

#### 3.1.1 Evidence from Pre-Clinical Studies

Several studies documented the effect of flaxseed oil in animal models of hypertension showing its anti-hypertensive potential; some of them also proposed molecular mechanisms of lowering systolic BP by flaxseed oil. Sekine* et al*. [65] indicated that the levels of plasma vasodilators (bradykinin, prostaglandin I2 and NO metabolites) were significantly increased in SHR while vasoconstrictors (angiotensin II (AngII) and thromboxane A2) did not change. Further, the application of a diet enriched with 10% flaxseed oil (4 weeks) reduced angiotensin-converting enzyme (ACE) mRNA expression and ACE activity in the aorta [66]. However, this BP-lowering mechanism of flaxseed oil in SHR is not associated with improved endothelium-dependent vasorelaxant response in the aortic rings; no changes in vascular morphology or increased sensitivity to NO were observed [67]. Mechanisms underlying the vascular effects of flaxseed oil were investigated also in isolated aortic rings in the presence/absence of endothelium bringing controversial results. In the presence of endothelium, flaxseed oil treatment increased vascular reactivity to phenylephrine through Reactive Oxygen Species (ROS) production and Cyclooxygenase-2 (COX-2) derived Tromboxane A2 (TXA2) production in the group; however, the treatment didn’t worsen the endothelium-dependent relaxation via acetylcholine. Endothelium removal increased the response to phenylephrine, but this effect was reversed by flaxseed oil application, suggesting that flaxseed oil treatment possibly can reduce negative endothelial modulation [68]. Regardless, there is still clear evidence of the effectiveness of flaxseed oil as an antihypertensive formula based on measuring BP in an animal model of metabolic syndrome [69]. In studies exploring the effects of flaxseed oil in Sprague-Dawley (SD) rat offspring, a flaxseed oil-rich diet was administered to mothers one week before mating with males (fed by flaxseed oil-rich diet too), during pregnancy (3 weeks), lactation (3 weeks) and further to pups until 30 weeks of age and hypertension was induced by feeding the animals with high dietary casein content (30%). In the results, hypertension development was inhibited in flaxseed oil-fed groups compared to α-linolenic acid (ALA)-deficient (10% safflower oil) group and/or high protein content (20 or 30% casein) ALA-deficient group [70, 71]. Following a 4-week feeding with a diet enriched with 10% flaxseed oil and/or exercise in a genetically modified obese Zucker rat strain, reduced BP was observed in both flaxseed oil-fed and exercise groups. Moreover, the combination of endurance exercise and a flaxseed oil diet resulted in an even greater improvement in mean arterial BP [72]. Recently, the antihypertensive effect of flaxseed oil administration for 21 days was demonstrated in deoxycorticosterone acetate-salt (DOCA-salt) rats. The flaxseed oil diet suppressed the development of hypertension from the early phase (day 7) until the end of the experiment (day 21) in both low-dose and high-dose flaxseed oil groups. Moreover, flaxseed oil significantly suppressed activation of ACE in kidneys [73].

**Fig. 2.**
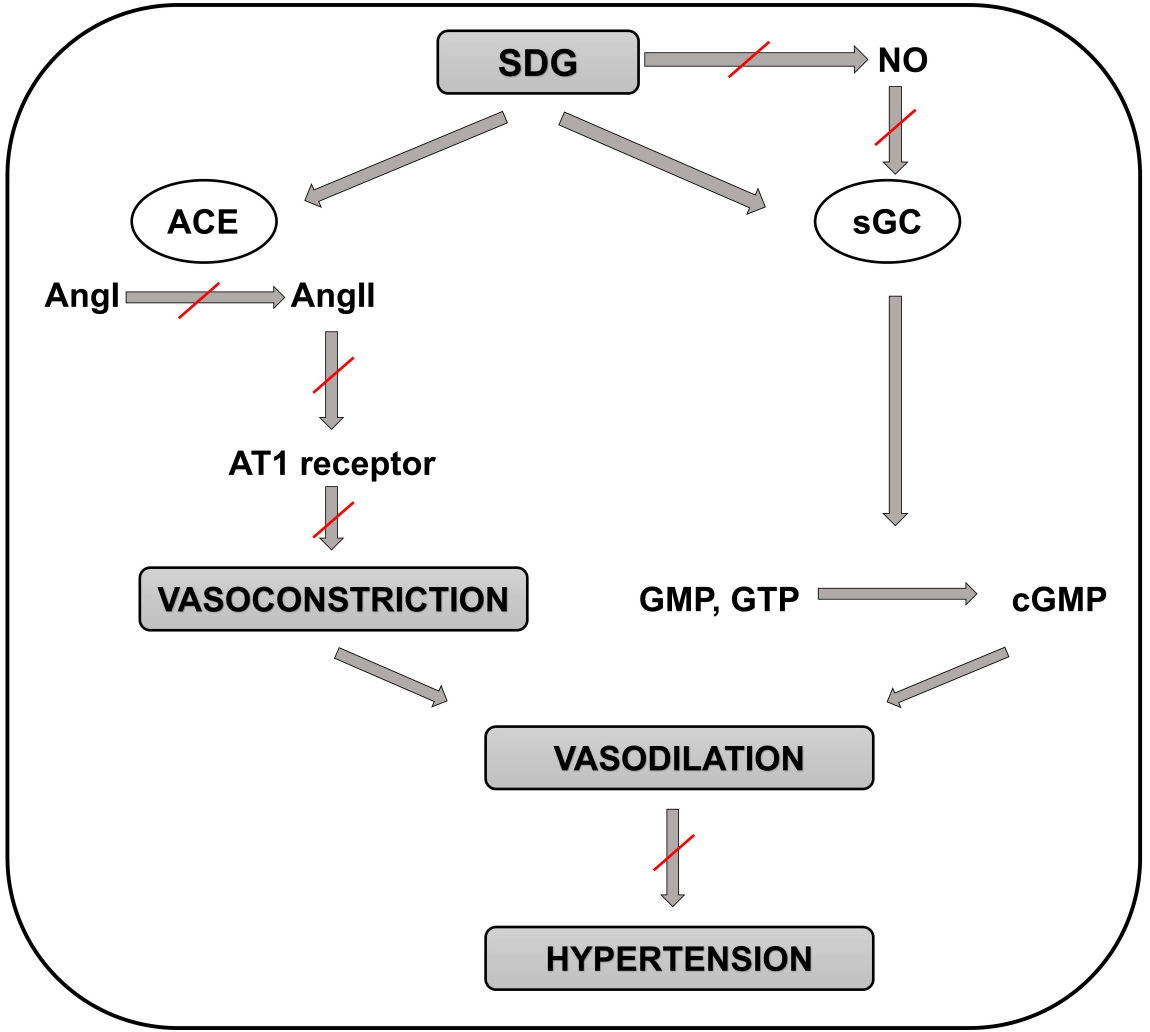
**Schematic representation of the molecular mechanisms involved in the positive effects of SDG in preventing the development of hypertension**. Positive effect of SDG on preventing hypertension is not mediated by NO production but through the guanylate cyclase enzyme. Then guanylate cyclase catalyzes the reaction of guanosine triphosphate (or guanosine monophosphate) to $3^{\prime }$,$5^{\prime }$-cyclic guanosine monophosphate (cGMP) which ultimately leads to vasodilation. Another proposed pathway of SDG effect on blood pressure is the inhibition of ACE resulting in decreased conversion of angiotensin I to angiotensin II. Then angiotensin II, which is a powerful vasoconstrictor through its binding to angiotensin II (AT1) receptor, could not cannot execute its function. This process also leads to vasodilation thus ameliorating the development of hypertension. ACE, angiotensin-converting enzyme; AngI, angiotensin I; AngII, angiotensin II; AT1 receptor, angiotensin II receptor type 1; cGMP, $3^{\prime }$,$5^{\prime }$-cyclic guanosine monophosphate; GMP, guanosine monophosphate; GTP, guanosine triphosphate; NO, nitric oxide; SDG, secoisolariciresinol diglucoside; sGC, soluble guanylate cyclase enzyme.

Animal studies evaluating effects of the major polyphenol contained in flaxseed oil, SDG, on BP demonstrated very similar results as the flaxseed oil itself. Prasad* et al*. [74] found that small doses of SDG (3 and 5 mg/kg) caused dose-dependent decreases in the systolic, diastolic, and mean arterial pressures in rats which probably are not mediated via NO signaling despite these BP-lowering effects might be due to guanylate cyclase activation. Further, higher dose of SDG (10 mg/kg) caused significant decreases in the arterial pressures with more pronounced antihypertensive effect on diastolic pressure in rats, likely via ACE/AngI/AngII inhibition [75] (Fig. 2). Finally, SDG (25 mg/kg) exerted antihypertensive effects in an animal model of monocrotaline-induced pulmonary arterial hypertension (PAH) in rats likely via improving redox homeostasis [76].

#### 3.1.2 Evidence from Clinical Studies

In a randomized controlled interventional trial with 60 patients with metabolic syndrome receiving a daily dosage of 25 mL of flaxseed oil for 7 weeks, lower systolic BP and diastolic BP and reduced levels of malondialdehyde as oxidative stress biomarkers were detected in the treated group. On the other hand, no difference in blood lipid levels and fasting blood sugar were shown in a flaxseed oil-treated group [77]. Interestingly, a 24-weeks of natural flaxseed oil blended with palm oil administration had beneficial effect on blood pressure in subjects with essential hypertension and coronary artery disease [78]. In a double-blinded randomized placebo-controlled study, a high intake of flaxseed oil for 12 weeks had an effect on reducing the levels of plasma fatty acid content and TNF-α marker, but no effect on vascular function during fasting and postprandial phase in untreated pre-hypertensive and stage I hypertensive obese and overweight individuals [79]. Finally, in a randomized, double-blinded placebo-controlled study, 44 patients with coronary artery disease received 5 g of flaxseed oil in 200 mL of 1.5% fat milk for 10 weeks. At the end of the intervention, a significantly reduced level of triacylglycerides (TAG) and diastolic BP was revealed, but other plasma lipid parameters remained unaffected by flaxseed oil [80].

### 3.2 Effects of Flaxseed Oil on Atherosclerosis

#### 3.2.1 Evidence from Pre-Clinical Studies

Several studies documented potential effects of flaxseed oil on the development of atherosclerosis, a disease tightly related to changed levels of plasma TAG, lipoproteins and also inflammation which occurrence is high especially in cardiometabolic diseases, e.g., obesity or metabolic syndrome. Important insight into potential anti-atherogenic effect of flaxseed oil brought a study exploring effects of flaxseed oil on aortic remodeling associated with diabetes during pregnancy in adult offspring. Diabetic female rats were fed with a flaxseed oil-enriched diet during pregnancy and lactation for 21 days, thus male and female pups were maintained on a standard diet until 180 days. The application of flaxseed oil preserved male offspring aorta elastic fibres deposition and improved the thickness of aorta intima-media layer in offspring of both genders [81]. Potential anti-atherogenic effect of flaxseed oil suggested also a study documenting significantly reduced plasma TAG and non-esterified fatty due to flaxseed oil treatment in male Wistar rats with metabolic syndrome [69]. Dietary flaxseed oil was shown to be beneficial by inhibition of macrophages cell foam formation in a Peroxisome proliferator-activated receptor-γ (PPARγ)-dependent manner via its main ω3 fatty acid metabolite, 12-hydroxyeicosapentaeonic acid [82]. In the high fat diet-induced atherosclerotic Apolipoprotein E (ApoE)-/- knockout mice after 12 weeks of flaxseed oil intervention, reducing of atherosclerotic lesions was observed [83]. Based on the fact that atherogenesis is connected with endoplastmatic reticulum stress and metabolic imbalance, a study monitoring an activation of G-protein coupled receptor 120 (GPR120) (an early inflammatory and endoplasmic reticulum (ER)-stress marker) in aorta of two different mice strains was performed. In a Swiss obese-prone mice, but not in LDLr-KO atherosclerotic-prone mice, flaxseed oil induced a partial activation of GPR120. The blood lipid profiles were improved in both animal models after receiving flaxseed oil enriched diet [84]. Moreover, treatment with the main flaxseed oil polyphenol SDG decreased levels of TAGs, total cholesterol (TC) and LDL-C and increased vessel density and heme oxygenase-1 (HO-1), and up-regulated expression of vascular endothelial growth factor (VEGF) and phosphorylated endothelial nitric oxide synthase (p-eNOS) in male SP rats with diet-induced hypercholesterolemia [85].

#### 3.2.2 Evidence from Clinical Studies

Human studies aimed at evaluating anti-atherogenic potential of flaxseed oil were mostly based on measuring the plasma lipid profile or markers of oxidative stress and inflammation as the indirect predictors of atherogenic risk. These studies brought inconsistent results. Despite a double blinded trial in 56 participants without coronary heart disease brought a clear evidence that daily consumption of flaxseed oil capsule (5.2 g) increased the plasma concentrations of cardioprotective (n-3) FA in humans [86], the same flaxseed oil capsule supplementation did not affect plasma lipoprotein concentration or particle size and increased circulating TC levels in human subjects [87]. In addition, a 12-weeks supplementation with 2 g flaxseed oil capsule exerted no effect on the plasma levels of TC, HDL-C, LDL-C and triglycerides (TG) in 86 healthy males and females in a double blinded, placebo controlled clinical study [88]. Surprisingly, a randomized, double-blinded, crossover study in 15 individuals receiving 10 g of flaxseed oil for 12 weeks showed significantly reduced levels of small density LDL, especially in subjects with TAG concentrations higher than 100 mg/dL [89]. Inflammatory conditions represent additional risk factor for the development of atherosclerosis. It was documented in 48 healthy subjects that a daily intake of 2 g flaxseed oil for 12 weeks reduced levels of inflammatory markers vascular cell adhesion molecule 1 (VCAM-1) and soluble E-selectin [90]. Similarly, 8-week treatment with flaxseed oil significantly reduced soluble VCAM-1 and systemic inflammation marker high sensitivity C-reactive protein (CRP) [91]. In addition, 6-week administration of 500 mg of SDG (main phenolic component of flaxseed oil) to 22 postmenopausal women had an anti-inflammatory effect documented by reduced CRP concentration; however, no significant differences in plasma concentrations of interleukin-6 (IL-6), TNF-α, VCAM-1 and monocyte chemoattractant protein-1 (MCP-1) were observed [92].

### 3.3 Effects of Flaxseed Oil in Other Cardiovascular Diseases

#### 3.3.1 Evidence from Pre-Clinical Studies

Since CVD are the major consequences of metabolic syndrome, a study comparing effects of flaxseed oil (rich in α-linolenic acid — C18 ω-3 PUFA) with effects of linoleic acid (C18 ω-6 unsaturated acid) and oleic acid (C18 monounsaturated acid) in a diet-induced animal model of metabolic syndrome was performed and it was found that flaxseed oil improved left ventricular structure and function and reduced diastolic stiffness [69]. The effects of 4-week oral pretreatment with flaxseed oil (0.4 g/kg/day) on isoproterenol-induced myocardial infarction was investigated in male Wistar rats. Even though no significant effect of flaxseed oil was examined on serum levels of creatine kinase-myocardial band (CK-MB), the histopathological examination revealed significantly reduced heart tissue destruction and decreased CaCl-2-induced mitochondrial swelling [93]. On the other hand, 28 days of flaxseed oil (1.9% w/w) administration lowered the levels of two strong cardiac markers — cardiac Troponin I (cTnI) and lactate dehydrogenase (LDH) in the same protocol of myocardial infarction in Sprague-Dawley rats [94]. Possible gender-dependent effect of 6-week administration of flaxseed oil (300 mg/kg/day) on cardiac function was found in study using adult Wistar rats of both genders manifested by mild improvement of the isolated myocardium function in male, but not in female rat hearts [95]. In an effort to prevent cardiac pathological remodeling post-Myocardial Infarction (post-MI), milled flaxseeds, flaxseed oil or flaxseed lignan SDG were administered to rats for 10 weeks (2 weeks before and 8 weeks after coronary artery ligation). Cardioprotective anti-remodeling effect was observed only in the flaxseed oil-treated group manifested by decreased collagen 1 expression which was associated with upregulation of potentially cardioprotective micro RNAs (miRNAs) (miR-133a, miR-135a, miR-29b) [96].

Regarding effects of SDG, a major polyphenol enriched in flaxseed oil, several studies documented its beneficial cardiovascular effects in animal models. SDG has been shown to decrease infarct size and improve left ventricular functions (increased ejection fraction, fractional shortening and reduced inner diameter in systole) and improve capillary and arteriolar densities in male SD rats with diet-induced hypercholesterolemia [85]. SDG treatment (2 weeks) also reduced infarct size in Langendorff-perfused isolated rat hearts exposed to 30-min global ischemia and 120-min reperfusion, and this was associated with decreased cardiomyocyte apoptosis, increased protein expression of VEGF, angiotensin-1 and p-eNOS. Moreover, SDG improved myocardial function evidenced by increased capillary density and improved ejection fraction *in vivo * [97].

#### 3.3.2 Evidence from Clinical Studies

Regarding clinical evidence of potential beneficial effects of flaxseed oil in cardiovascular disease, antioxidant and anti-inflammatory potential of flaxseed oil was documented in a human study in type 2 diabetic patients with coronary heart disease. Supplementation with 1000 mg of omega-3 FA from flaxseed oil twice a day for 12 weeks reduced levels of insulin and CRP and increased total antioxidant capacity in the blood [98].

## 4. The Effect of Soybean Oil on Cardiovascular Health and Diseases

Soybean oil is considered one of the most edible oils worldwide (approximately 30% of oil’s consumption) with promising ratio of low saturated FA content vs. high amount of polyunsaturated FA content, what makes it one of the most common sources of omega-3 and 6 FA. In addition to high content of unsaturated FA, soybean oil might be beneficial for cardiovascular health due to its other important components — polyphenols, whose representation compared to other vegetable oils, is quite high. The most enriched polyphenols in the soybean oil are phenolic acids. Phenolic acid content in soybean oil is composed of p-hydroxybenzoic acid, vanillic acid [99, 100], caffeic acid [101], p-coumaric acid [102, 103], ferulic [104, 105] and sinapic acid [106, 107]. Many of these phenolic acids have been proven to exert several cardio- and vasculo-protective effects, including cardioprotective, vasorelaxant, anti-inflammatory, or antioxidant [100, 102, 103, 104, 105, 106, 107, 108, 109]. Thus, beneficial effects of soybean oil could be attributed not only to its lipid composition but potentially also to its polyphenolic content.

### 4.1 Effects of Soybean Oil in Atherosclerosis

#### 4.1.1 Evidence from Pre-Clinical Studies

Extensive research has been performed exploring potential anti-atherogenic effects of dietary soybean oil, usually compared to other frequently used food oils. It has been shown that 6-week administration of high-fat diet containing 14% of soybean oil to male hamsters significantly reduced levels of serum TC, LDL-C and LDL-C/HDL-C ratio (this ratio represents so called “Atherogenic Index” [110] and increased levels of HDL-C compared to diet containing the same content (14%) of palm oil, suggesting potential anti-atherogenic effect of soybean oil. On the other hand, soybean administration significantly elevated serum thiobarbituric acid reactive substances (TBARS) levels when compared to palm oil-treated group, suggesting enhanced oxidative stress likely due to higher FA-unsaturation of soybean oil [111]. Surprisingly, a mixture of soybean oil and lard administered to male C57BL/6 J mice for 12 weeks resulted in a significantly lower levels of LDL-C and TC and higher levels of HDL-C compared to lard or soybean oil alone. Only levels of TAG were reduced after soybean oil administration compared to other groups [112]. In addition, soybean oil was used as control oil in a study assessing the effects of conjugated linoleic acid (CLA)-enriched ghee (clarified butter originating from India used for cooking, as a traditional medicine, and for religious rituals) as a potential anti-atherogenic food in a female Wistar rats. Feeding the animals for 16 weeks with a diet enriched by 200 g/kg of soybean oil and 200 g/kg of CLA ghee resulted in significantly reduced levels of TC and TAG in serum and aorta and increased serum HDL in a CLA ghee group compared to soybean oil group suggesting the anti-atherogenic potential of soybean oil lower than anti-atherogenic potential of CLA ghee [110].

On the contrary, there are also studies describing pro-atherogenic effects after excessive consumption of soybean oil. It was shown that treatment of rats with high fat diet containing 20% of soybean oil elevated levels of serum plasma lipids similarly as treatment with lard. Moreover, soybean oil increased systolic arterial pressure, oxidative stress and also showed pro-inflammatory potential proven by increased plasma myeloperoxidase activity [113]. In addition, dose-dependent pro-atherogenic effect of soybean oil was shown in C57BL/6 mice after 1-month administration of soybean oil-based emulsion (80/160 mg/mouse/day) manifested by increased lipid peroxidation and lipid accumulation in aortas and serum [114].

Other studies used various approaches to investigate effects of soybean oil on atherosclerosis. In a study comparing effects of conventional versus modified soybean oil, a Western diet enriched with 5% (w/w) conventional (containing n-6 PUFA linoleic acid) or modified (enriched in n-9 MUFA oleic acid) soybean oil were administered to LDL receptor knock-out mice for 12 weeks. Although a diet containing conventional soybean oil decreased plasma lipid levels (TC, LDL and very-low-density lipoprotein (VLDL)), it had no effect on atherosclerotic plaque size. On the other hand, modified soybean oil suppressed size of atherosclerotic plaques, but had no effect on plasma lipid levels [115]. Sung* et al*. [116] explored anti-atherogenic potential of soybean oil mixed with medium chain triglycerides (MCT) vs. regular soybean oil in an animal model of streptozotocin-induced type 2 diabetes. After 8 weeks of receiving high fat diet (254.4 g soybean oil/kg or 127.2 g of soybean oil + 137.9 g MCT oil), the lipid profile was better in soybean/MCT oil-treated group (lower levels of serum LDL-C and non-esterified FA, increased levels of HDL-C and HDL-C/LDL-C ratio) in comparison to regular soybean oil-treated group suggesting higher atherogenic risk in regular soybean oil group than in MCT group [116]. A well-known fact of negative impact of trans FA from oxidized vegetable oils and its association with higher risk of CVD led to a study investigating the effect of oxidized soybean oil vs. margarine on blood lipid levels, coronary artery lesions and coronary FA distribution in male rats were fed with high fat diet containing 20% of fresh soybean oil or 20% of oxidized soybean oil or 20% margarine for 4 weeks. Oxidized soybean oil as opposed to fresh equivalent resulted in elevated plasma lipids (TAG and LDL-C), and margarine even worsened those parameters. The same trend was observed in structural changes of the coronary arteries — diet rich in oxidized soybean oil negatively altered the structure less then margarine, but more than fresh soybean oil. Compared to low fat diet, fresh as well as oxidized soybean oil revealed fat droplets accumulation in the walls of coronary arteries [117]. Da Silva Alencar* et al*. [118] documented that increased involvement of soybean oil in a diet for 90 days decreased atherogenic index in healthy gilts. Finally, a reduced atherogenic index after 2-months of soybean oil-supplemented diet (15% w/w) administration was noted in bilateraly ovariectomized Sprague-Dawley rats [119].

#### 4.1.2 Evidence from Clinical Studies

Regarding the clinical research, potential pro-/anti-atherogenic effects of soybean oil could be assessed mostly by its effect on the plasma lipids/atherogenic indexes. In this line, 10-week soybean oil administration led to a significant reduction of TC and small dense LDL-C (sdLDL-C), but also to decreased HDL-C in hypercholesterolemic women. Moreover, the atherogenic indexes were better (lower) after rice bran oil or rice bran/palm oil mixture administration when compared to soybean oil suggesting higher anti-atherogenic potential of these oils than potential of soybean oil [120]. In a randomized controlled trial performed in healthy subjects with moderately elevated levels of LDL-C the effects of high oleic soybean oil as an alternative to unhealthy partially hydrogenated oils were analyzed and compared to effects of to other food oils such as palm oil, palm kernel oil or soybean oil. After 29 days, volunteers with high oleic soybean oil-enriched diet showed reduced LDL-C as well as reduced TC/DL and LDL/HDL ratios showing that high oleic soybean oil beneficially affect lipid and lipoprotein profiles associated with reduced CHD risk. On the other hand, this diet had minimal or no effect on markers of inflammation, lipid oxidation, hemostatic factors, blood pressure, and body composition [121]. Finally, acute consumption of soybean oil reduced levels of serum inter-cellular adhesion molecule 1 (ICAM-1) and TNF-α, the molecules implicated in atherogenesis, but had no effect on another atherogenesis-associated molecule VCAM-1 in healthy volunteers [122].

### 4.2 Effects of Soybean Oil in Hypertension

#### 4.2.1 Evidence from Pre-Clinical Studies

Only a limited number of studies focused on investigating the effects of soybean oil in hypertension. Papazzo* et al*. [123, 124] analyzed effects of 50-day application of canola (10% wt/wt) or soybean oil (10% wt/wt) in the absence or excess of salt intake in spontaneously hypertensive stroke prone rats (SHRSP). In the presence of salt in diet, BP was elevated in both canola and soybean oil groups in comparison to a soybean oil group without salt. However, in the soybean oil group without salt, increased level of red blood cells (RBC) glutathione peroxidase (GPx) and decreased levels of HDL-C and RBC malondialdehyde (MDA) were detected compared to canola or soybean oil group with salt in diet. In the absence of excessive salt in diet, decreased BP, decreased levels of LDL-C and TC and increased levels of RBC GPx and RBC superoxide dismutase (SOD) were found in a soybean oil group when compared to a canola oil group without salt intake suggesting anti-hypertensive, antioxidant and anti-atherogenic effects of soybean oil [123]. In a second study, lower BP, elevated plasma MDA and TAG and decreased LDL-C were found in soybean oil group compared to canola oil group, both with excess of salt intake confirming the anti-hypertensive potential of soybean oil in salt-induced hypertension [124]. Moreover, without salt intake soybean oil decreased contractile response to norepinephrine as compared to canola [124]. Considering everyday use of soybean oil for cooking especially in Asia, the effects of 5-times or 10-times overheated soybean oil compared to fresh soybean oil (all 15% w/w) on BP, vascular properties and inflammation were investigated in male SD rats. Results were coherent as in both groups using overheated soybean oil elevated BP, increased aortic wall thickness and circumferential wall tension as well as increased levels of plasma TXA2/Prostaglandin I2 (PGI2) ratio, endothelial VCAM-1, ICAM-1 and LOX-1 were found as opposed to fresh soybean oil [125].

#### 4.2.2 Evidence from Clinical Studies

In a randomized, double-blinded placebo-controlled clinical study in metabolic syndrome patients, 30-day soybean oil supplementation had no impact on blood pressure [109]. The same results (no changes in blood pressure) were observed in healthy volunteers receiving soybean oil for 35 days [126].

### 4.3 Effects of Soybean Oil in Other Cardiovascular Diseases

#### 4.3.1 Evidence from Pre-Clinical Studies

While previous studies were focused more on vascular effects of soybean oil, the following studies targeted its effects on the heart and cardiometabolic parameters. Specific approach took a study evaluating the effect of lipid emulsion Intralipid (n6 fatty acid-containing soybean oil-based emulsion) in comparison to another emulsion Omegaven (n3 fatty acid-containing fish oil-based emulsion) in intact beating perfused rat hearts specifically revealing insulin signaling and glucose uptake. The results turned in favor of Omegaven as opposed to Intralipid since Omegaven didn’t induce insulin resistance while Intralipid administration markedly diminished the insulin response. Moreover, it was identified that Omegaven preserved insulin signaling and supported glucose uptake and glycolysis via PP2A-Akt-PFK signaling pathway [127]. Considering that soybean oil is rich in ω-6 PUFA well known for its pro-inflammatory effects, a study using soybean oil as a negative control to fish oil was designed to examine the effects of both oils on the inflammation in ischemia-induced injury in the male Wistar rat hearts. After 20 days of pretreatment with fish or soybean oil daily by gavage (3 g/kg/day) before inducing ischemia by ligation of the left coronary artery, hearts were better preserved by fish oil against dysfunction in comparison to soybean oil. This was demonstrated based on echocardiographic analysis after heart infarction showing decreased infarct size and improved left ventricular function in the fish oil group as well as decreased left ventricular cytokine-induced neutrophile chemoattractant 2 α/β (CINC 2 α/β), interleukin 1β (IL1β) and TNFα levels, increased left ventricular adenosine triphosphate (ATP) levels, reduced creatine kinase and caspase-3 activities and decreased coronary blood flow [128]. Miralles-Pérez* et al*. [129] assessed the effects of an increased concentration of DHA (80%) in fish oil compared to other edible oils (one amongst them a soybean oil) administrated for 10 weeks in a dose of 0.8 mL/kg/day on cardiometabolic parameters in healthy male rats. When compared to soybean oil, fish oil containing 80% of DHA reduced plasma TC and plasma fat content; on the other hand, it was accompanied with higher lipid peroxidation and antioxidant response. In fact, lipid peroxidation profile revealed clear dependence on the degree of unsaturation of the oils — 80% DHA fish oil > EPA/DHA 1:1 fish oil > soybean oil > coconut oil. Nevertheless, soybean oil has shown the most beneficial LDL-C/HDL-C ratio compared to all other oils used in the study [129].

#### 4.3.2 Evidence from Clinical Studies

In relevant human studies investigating the effects of soybean oil on cardiovascular health, soybean oil was mostly included only as a control group; moreover, its effects were usually not as good, or even worse, than effects of the main studied compound. For example, 12-week administration of Omega3Q10 formulation (composed of marine omega-3 poly-unsaturated FA) and soybean oil as a control, lowered chest tightness and palpitation were detected in Omega3Q10 group compared to soybean oil [130]. Differences in antioxidant capacity of soybean oil were detected after comparing it with either Brazil nut oil (10 mL/day) in patients with metabolic syndrome or with rice bran oil (30 mL/day) in hyperlipidemic patients delivered for 4 weeks. When compared to brazil nut oil, soybean oil improved antioxidant capacity demonstrated by Trolox equivalent antioxidant capacity (TEAC) assay [109], but it was significantly lower compared to rice bran oil, demonstrated by increased oxygen radical absorbance capacity (ORAC) and ferric reducing antioxidant power (FRAP) levels of rice bran oil [131]. Considering oxidative stress as one of the main risk factor for CVD, blood MDA levels were higher in soybean oil group compared to Brazil nut oil [109] or camellia oil [132]. Moreover, increased blood LDL-C and TC and decreased HDL-C were detected in soybean oil group as opposed to other oils used in particular studies (Camellia oil, Brazil nut oil, rice bran oil and Omega3Q10 formula) [109, 130, 131, 132].

Thus, soybean oil, despite it is one of the most widely used oil for cooking and despite its beneficial ratio of unsaturated/saturated FA and high content of phenolic compound, cannot be fully considered beneficial for cardiovascular health due to inconsistent and partially also controversial results of experimental studies. Finally, it should be noted that despite soybean oil is disposed of high content of total phenolic compound content, the investigation of its effects on cardiovascular health didn’t involve this fact so far, and attributed the effects of this widely used oil mostly on its FA content without special focus on the role of its phenolic acid content in its cardiovascular effects.

## 5. The Effect of Sesame Oil on Cardiovascular Health and Diseases

Sesame seed oil (obtained from Sesamum indicum) is popular oil consumed as traditional health food especially in India, China and other East Asian countries [133, 134]. This oil is rich not only in both monounsaturated and polyunsaturated FA (approximately 47% oleic acid and 39% linoleic acid) but also in phytosterols, high amounts of vitamin E (40 mg/100 g oil), methylenedioxyphenol derivatives and lignans (subgroup of polyphenols) such as sesamin, episesamin, sesamol, sesamolin or sesamolinol [133, 135, 136, 137, 138]. The total plant lignan concentration in sesame seed (2180 µmol/100 g) is much higher than in flaxseed (820 µmol/100 g). The concentration of the most abundant isomer in sesame seed, the sesamin is 1520 ± 6.8 µmol/100 g [139]. Raw sesame oil contains 0.5–1.1% sesamin, 0.2–0.6% sesamolin and trace amounts of sesamol [140].

Sesame oil is well known for its multiple beneficial properties: antioxidant [141], anti-inflammatory [142], blood sugar-controlling [143], plasma cholesterol, LDL-C and TG-lowering [144], anti-arthritic [145], wounds and burns-healing [146], hair and skin-repairing [147], and many others. Regarding cardiovascular health, sesame oil possesses anti-hypertensive, anti-atherogenic, anti-inflammatory and cardioprotective effect.

### 5.1 The Effect of Sesame Oil in Hypertension and Heart Hypertrophy

#### 5.1.1 Evidence from Pre-Clinical Studies

A few studies examined the effect of sesame oil on BP in animal models of hypertension documenting its BP reducing effects. Liu* et al*. [148] showed that sesame oil administered by oral gavage (0.5 or 1 mL/kg/d for 7 days) effectively reduced the systolic and diastolic BP and also positively altered electrocardiogram (ECG) (reduced QRS duration, PR and QT intervals) in a model of hypertensive (DOCA/salt) uninephrectomized male SD rats. In the same study sesame oil also decreased the heart mass, left ventricle thickness and the diameter of cardiomyocytes, suggesting the regression of left ventricular hypertrophy due to feeding with sesame oil.

Later, the study by Liu and Liu [149] also proved that sesame oil significantly decreased the size of cardiomyocytes in DOCA salt rats, and additionally it also decreased the levels of cardiac renin, angiotensin-converting enzyme and AngII, down-regulated the expression of angiotensin type 1 receptor, c-Jun N-terminal kinase (JNK) and p38 Mitogen-Activated Protein Kinase (MAPK) and apoptosis signal regulating kinase 1, c-Fos and c-Jun in DOCA salt hypertensive rats.

Positive effect of 9-week feeding with sesame oil (in normal and high-cholesterol diets (200 g/kg)) has been shown also in in anesthetized SHR rats by reducing systolic BP; however, other pressure parameters such as diastolic BP, mean arterial BP and arterial pulse pressure did not change. In contrary, in conscious SHR rats, no changes in resting BP or other pressure parameters in sesame oil group compared with control have been documented. In both anesthetized and conscious SHR rats sesame oil significantly increased heart rate (to >400 beats/min) indicating tachycardia [150].

Animal studies evaluating effects of the major polyphenol contained in sesame oil, sesamin, on BP demonstrated very similar results as the sesame oil itself. Multiple studies proved anti-hypertensive effect of sesamin in different treatment modes and different models of hypertension in rats suggesting that sesamin could play a key role in anti-hypertensive effect of sesame oil. For example, application of sesamin to hypertensive DOCA salt rats (diet containing 1% sesamin for 4 or 5 weeks) decreased BP, left ventricle weight, wall thickness, wall area and the wall-to-lumen ratio of aorta and superior mesenteric artery [151, 152]. Nakano* et al*. [153, 154] observed that sesamin (containing diets 0.1 or 1 w/w%, for 5 weeks) significantly suppressed the development of DOCA-salt-induced hypertension, moreover sesamin was able to suppress the production of aortic superoxide, improved the DOCA-salt-induced impairment of endothelium-dependent relaxation, abolished the increase in nicotinamide adenine dinucleotide phosphate (NADPH) oxidase activity and suppressed increases in p22phox, gp91phox and Nox1 mRNA expression. Matsumura* et al*. [155] studied anti-hypertensive effect of sesamin (1 w/w% in commercial normal diet for 19 or 24 weeks) in salt-loaded group and an unloaded group of SHRSP. They found that sesamin significantly suppressed the development of hypertension, lowered the left ventricle weight and decreased the wall thickness and wall area of aorta and superior mesenteric artery. This anti-hypertensive effect of sesamin was much more pronounced in salt-loaded SHRSP than in unloaded rats.

Recent studies offer an insight into the mechanisms of anti-hypertensive effect of sesamin. Kong* et al*. [156] demonstrated that treatment with sesamin (by gavage, 120 or 60 mg/kg/day for 8 weeks) in two-kidney, one-clip renovascular hypertensive rats fed with a high-fat, high-sucrose diet reduced SBP, improved acetylcholine-induced vasodilatation and enhanced NO activity in the thoracic aorta. Restoration of NO activity was associated with upregulation of eNOS, decreased malondialdehyde content and suppression of p47phox and nitrotyrosine protein expression. Later, the same authors [157] examined mechanisms involved in the effect of sesamin (by gavage, 160 or 80 mg/kg/day for 8 weeks) on aortic NO bioactivity in SHR. Sesamin treatment led to upregulation of p-eNOS, suppression of eNOS dimer disruption, reduced NO oxidative inactivation through downregulation of p47phox and amelioration of eNOS uncoupling. Levels of GPx and catalase activity did not change but total total SOD activity and Cu/Zn-SOD protein expression was reduced. These data confirmed anti-hypertensive and the endothelial function-protective effects of sesamin.

Finally, positive effect of sesamin (oral administration of 100 and 200 mg/kg body weight, for 4 weeks) on the blood pressure was documented in streptozotocin (STZ)-induced diabetic rats [158]. Sesamin not only reduced BP, but also improved blood glucose levels, body weight and heart rate and reduced QT interval in diabetic rats.

#### 5.1.2 Evidence from Clinical Studies

Clinical studies observed beneficial BP-reducing effects of sesame oil supplementation in medicated hypertensive or diabetic hypertensive patients (different amounts and time of application: 35 g of oil/day/person for 60 days — [159]; 35 g of oil/day/person for 45 days — [160]; 35 g of oil/day/person for 45 days — [133]; 35 to 40 mL/person/d of blend 20/80% sesame/rice bran oil for 60 days — [161]; 30 mL/day in food for 8 weeks — [162]). In contrary, a very recent study by Moghtaderi* et al*. [163] did not prove positive effect of sesame oil on BP; however, in this study the exact amount of consumed oil in patients’ food was not specified, only the time of consumption (9 weeks). Positive effect of sesame oil (or sesamin) consumption on hypertension and endothelial function was confirmed in a clinical study of Karatzi* et al*. [164] where the effects of sesame oil (35 g/day) on endothelial function were investigated in two phases: in the postprandial state (12 hour fast and 2 hours after consumption) and after long-term consumption (2 months). Both acute and long-term consumption of sesame oil had positive effect on the endothelial vasodilatory capacity, assessed by flow-mediated dilatation while beneficial effect of sesame oil on the inhibition of endothelial activation assessed by ICAM-1 levels was found only after long-term consumption of sesame oil. In addition, it was shown that polyphenol sesamin could be a key (important) substance of BP-reducing effect of sesame oil. A clinical study of Miyawaki* et al*. [165] demonstrated that after 4 weeks administration of 60 mg sesamin BP significantly decreased by an average of 3.5 mmHg systolic BP and 1.9 mmHg diastolic BP.

### 5.2 The Effect of Sesame Oil in Atherosclerosis

#### 5.2.1 Evidence from Pre-Clinical Studies

Several animal studies investigated the effect of sesame oil on the atherosclerosis. Bhaskaran* et al*. [166] demonstrated beneficial effect of sesame oil consumption (atherogenic diet with sesame oil, 170 g/kg for 12 weeks) manifested by the reduction of the atherosclerotic lesion formation and plasma cholesterol, TG, and LDL-C levels in male LDLR-/- mice (LDL receptor knock-out mice with pre-existing atherosclerosis). The anti-atherosclerotic effect (reduced atherosclerotic lesions, plasma cholesterol, TG, and LDL-C levels) of sesame oil treatment was later proved also in female LDLR-/- mice. In addition, an anti-inflammatory effect of sesame oil with reduction in plasma inflammatory cytokines such as MCP-1, RANTES, interleukin-1α (IL-1α), IL-6, and Chemokine (C-X-C motif) ligand 16 (CXCL-16) was documented in the same study. Moreover, sesame oil-treated group also exhibited changes in genes involved in cholesterol transport and metabolism: *ABCA1, ABCA2, APOE, LCAT, and CYP7A1 * [167]. Based on these results, authors proposed the theory of three major mechanisms by which sesame oil could inhibit atherosclerosis: (1) reducing plasma cholesterol by accelerated its catabolism (through the oxidation of cholesterol by cholesterol-7a-hydroxylase or CYP7A1); (2) enhancing reverse cholesterol transport (RCT) mediated by scavenger receptor class B type 1 (SR-B1) and ATP-binding cassette (ABC) transporters (ABCA1 and ABCG1); (3) controlling mediators of inflammation. Further, it was investigated whether atherosclerotic effect of sesame oil is mediated by the FA components or by the non-saponifiable components [142].

Inflammation is one of the major mechanisms involved in the process of atherogenesis. In line with this, sesame oil aqueous extract (SOAE) prepared by a unique method to separate the nonlipid components of sesame oil was tested for its anti-inflammatory effects. Treatment with SOAE significantly reduced a number of inflammatory markers including Ccl2 or MCP-1, Ccl5 or RANTES, IL -1α, interleukin-1β (IL-1β) and TNFα, in both RAW 264.7 macrophages (monocyte/macrophage-like cells, originating from Abelson leukemia virus transformed cell line derived from BALB/c mice) (200/500 µg/mL SOAE) and mouse peritoneal macrophages (200 µg/mL SOAE). SOAE in the doses 50 and 100 µg/mL, respectively, also significantly reduced LPS- induced TNFα levels in mice. Moreover, also other targets involved in atherosclerotic disease such as colony stimulating factor 2 (Csf2), Nuclear factor kappa-light-chain-enhancer of activated B cells (NF-κB1) and matrix metalloproteinase 3 (MMP3) were inhibited by SAOE in macrophages. Furthermore, SOAE inhibited LDL and HDL oxidation by Cu, MPO, or MPO+tyrosine *in vitro*. These results confirmed the anti-inflammatory and antioxidant effects of SAOE. Finally, SOAE (100 and 300 µg/mL, in RAW cells) also affected lipid metabolism by regulating genes involved in RCT, such as ABCA1 and liver X receptors (LXRs, transcription factors involved in the ABCA1 induction). Altogether these findings confirmed the three major mechanisms by which sesame oil could inhibit atherosclerosis [142]. Later, it was demonstrated in conditions of high-fat diet that SOAE (340 mg/kg for 15-weeks) reduced atherosclerotic lesions, plasma cholesterol, and LDL-C levels, reduced inflammatory genes expression, increased expression of genes involved in cholesterol metabolism and reversed cholesterol transport in LDLR-/- mice [168]. Sesame oil (1% in diet) and SOAE (0.75 mg/mouse/day) post-treatment (for 1 month) reduced preexisting atherosclerotic lesions (induced by atherogenic diet for 3 months), reduced inflammatory gene expression and induced genes involved in cholesterol metabolism and RCT in LDLR-/- mice [169]. Similarly, 1-month SOAE pre-treatment prevented development of atherosclerotic lesions induced by 2 months atherogenic diet, reduced pro-inflammatory genes expression, plasma levels of TNF-α, IL-6, MCP-1 and VCAM1 in LDLR-/- mice [170]. Surprisingly, sesamol, sesamin or other lignans were not present in SOAE thus these polyphenols were not identified as anti-inflammatory components of SOAE; on the other hand, combination of methoxyphenol compounds (detected in SOAE) exerted anti-inflammatory properties what favorize these components as mediators of anti-inflammatory effects of SOAE [171].

In contrary to previous finding, numerous studies demonstrated anti-atherogenic effects of sesamol and sesamin. Chen* et al*. [172] found that sesamol supplementation (50 or 100 mg/kg via oral gavage for 16 weeks) markedly reduced atherosclerotic lesion size in aortic arch associated with reduced plasma L5 type of LDL (the most electronegative type of LDL, subfraction of LDL) in hamsters fed with high-fat diet. Sesamol also decreased the expression of the L5-induced lectin-like oxidized LDL receptor-1 (LOX-1), decreased phosphorylation of p38-MAPK and activation of caspase-3 and increased phosphorylation of eNOS and Akt. Recently, Wang* et al*. [173] proved that administration of sesamol (25 and 50 mg/kg, by oral gavage 3-times per week for 8 weeks) caused significant decrease in atherosclerotic lesions in aorta and carotid artery and also in malondialdehyde levels in the kidney, plasma, and carotid artery of ApoE–/– mice subjected to 5/6 nephrectomy (5/6 Nx). Sesamol (0.3–3 µM) also suppressed H2O2-induced oxidative stress likely via reduction of phospho-IKKα levels and inhibition of p53 and caspase-3 in human aortic endothelial cells (HAECs) [173].

In addition to sesamol, also sesamin showed beneficial effects in atherosclerosis. Wu* et al*. [174] proved that pretreatment of HAECs with sesamin (10 or 100 µM) or sesamol (100 µM) caused significant reduction (35 or 70% decrease; respectively 30% in sesamol) in TNF-α-induced expression of ICAM-1. They both caused decrease in human antigen R (HuR) translocation, the interaction between HuR and the 3’UTR of ICAM-1 mRNA and also reduced the binding of monocytes to TNF-α-stimulated HAECs. Sesamin alone downregulated extracellular signal-regulated kinase (ERK) 1/2 and p38-MAPK. Sesamin caused decrease in ICAM-1 expression also in aortas of apolipoprotein-E-deficient mice *in vivo*. Thus, potential mechanism by which sesamin prevented development of atherosclerosis may include TNFα-induced decrease of ERK/p38 phosphorylation, nuclear translocation of NF-κB p65 and cytoplasmic translocalization of HuR with consequent inhibition of ICAM-1 expression resulting in the reduction of leukocytes adhesion. On the other hand, Loke* et al*. [175] showed that sesamin (1.3 mg/d; 64-mg/kg, for 10 weeks) exerted no significant effects on atherosclerotic lesion formation in the ApoE -/- gene knockout mice.

Sesamin has been also identified as a potential candidate for a treatment of vascular smooth muscle cell (VSMC)-specific vascular diseases due to its protective effect against platelet-derived growth factor (PDGF)-induced activation of VSMC. Freise* et al*. [176] proved that (+)-episesamin and sesamin (5 or 10 µM) reduced basal and PDGF-BB-induced proliferation and migration of human, murine and rat VSMC. This effect was mediated by activation of MAPK and PI3K pathways and by induction of HO-1 expression. Sesamin and episesamin also blocked the stimulatory effects of PDGF-BB on activation of NF-κB and induction of gene expression and secretion of matrix metalloproteinase 2 and 9 (MMP2 and MMP9). Moreover, in the study by Han* et al*. [177] sesamin (1, 5, and 10 µM) inhibited PDGF-mediated proliferation of VSMC through upregulating p27KIP1, p21CIP1, p5, inhibition of cyclin E-cyclin-dependent kinase 2 (CDK2) and cyclin D1-CDK4 expression which arrests the cell cycle in G0/G1.

**Fig. 3.**
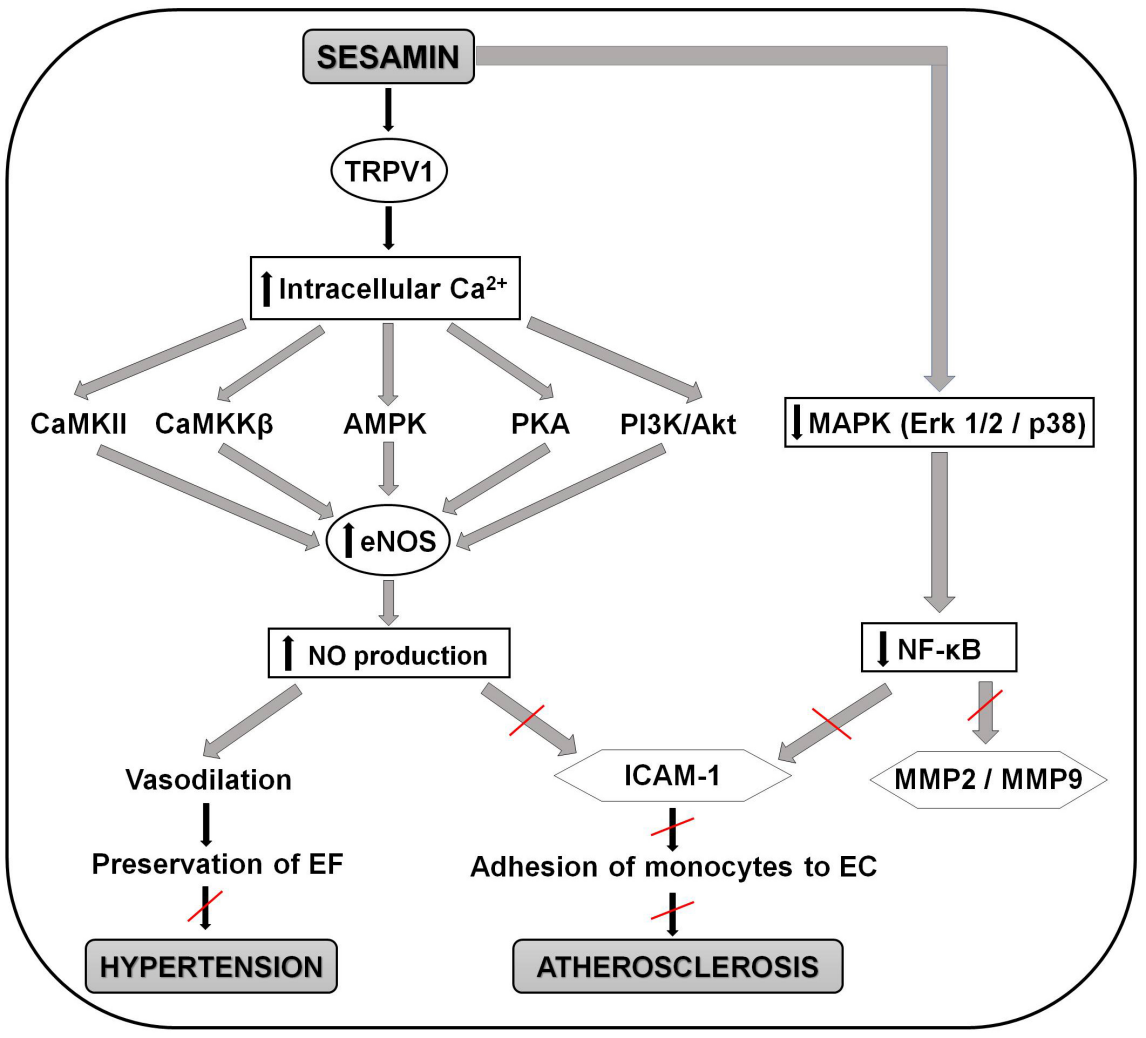
**Schematic representation of the molecular mechanisms involved in the positive effects of sesamin in preventing the development of atherosclerosis and hypertension**. Sesamin activates the TRPV1 channel and causes increase in intracellular calcium. This increase activates CaMKII, CaMKKβ, AMPK, PKA and PI3K/Akt signaling pathways, which induces eNOS activation and NO production. NO production leads to vasodilatation of the vessels, perseveration of the endothelial function and avoiding of the development of hypertension. Increase in NO production also inhibits the expression of ICAM-1. Another pathway of sesamin inhibition of ICAM-1 is via MAPK (Erk 1/2/p38) pathway. Sesamin downregulates extracellular signal-regulated kinase (ERK) 1/2 and p38 which cause decrease in nuclear translocation of NF-κB and then block gene expression and secretion of MMP2 and MMP9. Decrease in NF-κB also cause inhibition of ICAM-1 expression which avoids adhesion of monocytes to endothelial cells and prevents the development of atherosclerosis. AMPK, 5’ adenosine monophosphate-activated protein kinase; Ca, calcium; CaMKII, ${\mathrm{Ca}}^{2+}$/calmodulin-dependent protein kinase II; CaMKKβ, calcium ions/calmodulins stimulate protein kinase kinases β; EC, endothelial cells; EF, endothelial function; eNOS, endothelial nitric oxide synthase; Erk 1/2/p38, extracellular signal-regulated kinase 1/2/p38 mitogen-activated protein kinase pathway; ICAM-1, intercellular adhesion molecule 1; MAPK, mitogen-activated protein kinases; MMP2 and 9, matrix metalloproteinase 2 and 9; NF-κB, nuclear factor kappa-light-chain-enhancer of activated B cells; NO, nitric oxide, PI3K/Akt, phosphoinositide 3-kinases/ protein kinase b pathway; PKA, protein kinase A; TRPV1, transient receptor potential cation channel subfamily V member 1.

Recent study by Pham* et al*. [178] brings detailed insight into the molecular mechanisms behind the positive effect of sesamin on the endothelial function, anti-atherogenic and also anti-hypertensive effect. Sesamin (20 µM) caused increase in eNOS activation a NO production which may lead to vasodilatation, perseveration of the endothelial function and avoiding of the development of hypertension. It also suggested that sesamin cause increase in intracellular calcium via the transient receptor potential vanilloid type 1 (TRPV1) channel, an ion channel protein that can be activated by heat, protons, anandamide, and various ligands, and is responsible for increased calcium entry into the cells. Increase in intracellular calcium activates Ca2+/calmodulin-dependent protein kinase II (CaMKII), calcium ions/calmodulins stimulate protein kinase kinases β (CaMKKβ), protein kinase B (Akt kinase), 5’ adenosine monophosphate-activated protein kinase (AMPK), and protein kinase A (PKA) signaling pathways, which induced eNOS activation and NO production. Further, NO inhibits the TNF-α-stimulated expression of ICAM-1 and adhesion of monocytes to endothelial cells and prevent the development of atherosclerosis. The main pathways which participate in anti-atherogenic and anti-hypertensive effect of sesamin are summarized in Fig. 3.

#### 5.2.2 Evidence from Clinical Studies 

To our best knowledge, no clinical studies directly examined the effect of sesame oil, sesamol or sesamin on the development of atherosclerosis so far. The only one clinical study revealed the effect of sesame oil on the plasma lipid profile in hypercholesterolemic patients documenting decreased TG and LDL-C and increased HDL-C due to sesame oil supplementation which might be associated with anti-atherogenic effect of sesame oil. However, authors do not directly conclude these results as an anti-atherogenic effect of the sesame oil [144].

### 5.3 The Effect of Sesame Oil in Other Cardiovascular Diseases

Regarding the effects of sesame oil in other CVD than atherosclerosis, hypertension and cardiac hypertrophy, Saleem* et al*. [179] demonstrated positive effect of sesame oil (5 and 10 mg/kg) against doxorubicin-induced cardiotoxicity (decrease in necrosis, increase in the level of LDH, CK and aspartate transaminase (AST)) through the enhancement of endogenous antioxidants, reduction of lipid peroxidation and TNF-α in rat myocardium. Surprisingly, there are no experimental or clinical studies evidencing effects of sesame oil on ischemic heart disease, MI or cardiac ischemia-reperfusion (I/R) injury; however, there is a couple of studies documenting effects of sesamin and/or sesamol on experimental MI in rats [180, 181].

## 6. The Effect of Coconut Oil on Cardiovascular Health and Diseases

Coconut oil (obtained from coconut — Cocos nucifera) is also a mainstream popular oil highly recommended as a “Super food” product for better health in Western countries. Coconut or coconut oil originate from tropical and subtropical coastal regions (India, Indonesia, Philippines, Sri Lanka, Thailand, Malaysia) and it is used for centuries in the local diets (Ayurveda dates back coconut in diet up to 4000 years). In these countries, the coconut tree is also called the tree of life [182, 183]. Importantly, the process of production possibly influences the content of coconut oil. Original method of oil extraction from dried coconut meat (from copra) includes refining, bleaching, and deodorizing (RBD). More recent and more popular method is wet extraction, where the oil is extracted from fresh kernel of coconut by mechanical process (with or without heat) but without the process of RBD [184]. Coconut oil made by this process is defined as the virgin coconut oil (VCO) and involves higher concentrations (59.02% to 62.27%) of medium-chain FA (such as caproic acid, caprylic acid, capric acid, and lauric acid 50% of it) and higher concentration of total polyphenols (84 mg/100 g) and vitamin E (33 µg/100 g) [185, 186, 187, 188, 189].

Coconut oil is known for its numerous beneficial effects including prevention and treatment of Alzheimer disease [190], supporting bone health and preventing osteoporosis [191], controlling blood sugar [192], stress-reducing and antioxidant effects [193], preventing liver disease [194], reducing asthma symptoms [195], improving dental health [196] and reducing body weight [197]. On the other hand, there are also opposite or controversial opinions about the effects of coconut oil in the human diet due to high content of saturated FA (90%), which has been positively correlated with the increase of LDL-C [198]. Regarding this, health organizations such as World Health Organisation (WHO) or EFSA, made recommendations about its daily intake [199, 200]; e.g., WHO recommends limiting the intake of saturated fat to maximally 10% of the total daily calories [199]. In this review we summarize effects of coconut oil on cardiovascular health including its effects on hypertension, atherosclerosis, and its potential anti-inflammatory and cardioprotective effects.

### 6.1 The Effect of Coconut Oil in Hypertension and Heart Hypertrophy

#### 6.1.1 Evidence from Pre-Clinical Studies

Effects of coconut oil in hypertension have been studied in various animal models. Nurul-Iman* et al*. [201] demonstrated that 16-week coconut oil feeding in the dose 1.42 mL/kg (equal to one tablespoon (10 mL) which is the recommended daily minimum intake of the VCO in human) reduced the blood pressure in SD rats fed with five-times-heated palm oil (5 HPO, 15% weight/weight (w/w)). VCO also significantly increased the plasma NO levels (compared to 5 HPO group) and attenuated aortic rings vasoconstriction to phenylephrine without affecting vasorelaxation, thus improving endothelial function.

VCO in the same dose (1.42 mL/kg) also reduced cardiac lipid peroxidation (TBARS), decreased the activity of ACE and significantly prevented the increase in the myofibril width and area and nuclear size reduction compared to HPO group. The data suggested that the protective effect of the VCO is probably due to its high content of antioxidants (mainly phenols such as ferulic acid and p-coumaric acid) which might act against the harmful effects of HPO consumption [202]. VCO (200 g/kg, in diet for 16 weeks) reduced systolic BP also in male Wistar rats fed with high-carbohydrate diet [203]. On the other hand, Hamsi* et al*. [204] found no significant changes in blood pressure after treatment with fresh coconut oil (15% w/w for 24 weeks). Moreover, 5 and 10-times repeatedly heated (180 °C) coconut oil caused significant increase in BP and plasma thromboxane B2 (TXB2) and decrease in the plasma PGI2 levels. In the 10-times heated coconut oil group, no changes in plasma levels of VCAM-1, ICAM-1 and CRP were documented.

#### 6.1.2 Evidence from Clinical Studies

In line with findings from animal studies, two clinical studies demonstrated no significant effect of extra virgin coconut oil (EVCO, 50 g daily in usual diet for 4 weeks) or EVCO in capsules (10 mL/day = 10 capsules/day within the main meals) on systolic and diastolic BP both in normotensive [37] and hypertensive patients [205]. There were no changes in other parameters including body weights, Body Mass Index (BMI), central adiposity, fasting blood glucose and oxidative stress [37, 205].

### 6.2 The Effect of Coconut Oil in Atherosclerosis

#### 6.2.1 Evidence from Pre-Clinical Studies

Only a few relevant animal studies documented the effect of coconut oil on the development of atherosclerosis. Nevin and Rajamohan [206] investigated the effect of VCO feeding (10% w/w, in diet for 45 days) compared to copra oil (CO) and sunflower oil (SFO) on blood coagulation factors, serum lipid levels and *in vitro* LDL oxidation in cholesterol (1%) fed rat. Compared to CO and SFO, feeding with VCO significantly decreased blood coagulation and prevented atherosclerosis development. In addition, serum total cholesterol and TG, TBARS content of isolated LDL and erythrocyte membrane, thrombotic risk factors (platelets, fibrin, fibrinogen, and factor V), 6-ketoPGF1a and also hematological factors (white blood cells (WBC), hemoglobin (Hb) and RBC) were decreased in VCO-fed group compared to the other groups. Finally, the antioxidant vitamins levels (vitamin A and E) were higher in VCO group, and LDLs isolated from VCO-fed animals showed significant resistance to oxidation. Authors suggested that positive effects of VCO could be caused by unsaponifiable components of VCO such as vitamin E, provitamin A, polyphenols and phytosterols. This is in line with the previous studies which demonstrated that polyphenol fraction (PF) from VCO decreased *in vitro* oxidation of LDL [188, 207]. Positive effect of VCO on lipid peroxidation has been proven also by other studies [189, 208, 209, 210] which also demonstrated that supplementation with VCO (or PF) significantly increased antioxidant enzyme activities (levels of SOD, catalase (CAT), GPX and glutathione reductase (GR)) and prevented the oxidation of MDA, hydroperoxides (HP), conjugated dienes (CD) and protein carbonyls in serum and tissues (liver, kidney, heart) in various animal models. Comparing the CO and VCO has shown that VCO contain higher amounts of unsaponifiable components like polyphenols (84 mg per 100 g oil) and tocopherols (33.12 mg per 100 g oil) and polyphenols from VCO showed higher radical-scavenging activity [189, 208].

#### 6.2.2 Evidence from Clinical Studies

Regarding clinical evidence of the effects of coconut oil on the development of atherosclerosis it was demonstrated that the consumption of coconut oil (in a diet, 8 weeks) improved fat free mass, insulin sensitivity, increase plasma HDL-C and reduced plasma inflammatory markers such soluble vascular cell adhesion molecule 1 (sVCAM1) and MMP-9 in healthy men [211]. In addition, several clinical studies compared general health effects (including plasma lipid profile) of coconut oil with other food oils in various cohorts of human patiens; however, these studies were not directly focused on the effects of coconut oil on atherogenesis (for review see meta-analysis of clinical trials by Neelakantan* et al*. [212]).

### 6.3 The Effect of Coconut Oil in Other Cardiovascular Diseases

#### 6.3.1 Evidence from Pre-Clinical Studies

A few studies investigated the effect of coconut oil in other CVD and brought controversial or contradictory results. Isensee and Jacob [213] found that 10% hydrogenated coconut oil in diet (10 weeks) compared to another oils (corn, linseed and fish) had no significant effect on the size of the infarction and the incidence of ventricular fibrillation in male Wistar rats. Moreover, coconut oil consumption caused decrease in time between coronary occlusion and the first occurrence of extrasystoles. Muthuramu* et al*. [214] demonstrated that mortality rate after transverse aortic constriction (used for the induction of pressure overload-induced cardiomyopathy) was higher in coconut oil (CO) fed mice (10%, for 5 weeks) compared to standard chow-fed female C57BL/6 mice. In addition, CO caused increase in lung weight and had no effect on body weight gain and systemic insulin resistance. Moreover, feeding with CO caused decrease in myocardial capillary density, increase in interstitial fibrosis and worsened systolic and diastolic function in the pressure overload-induced cardiomyopathy. Mice fed with CO also showed higher myocardial glucose uptake, myocardial pyruvate dehydrogenase and acetyl-CoA carboxylase levels and lower myocardial TG and free FA. Finally, they CO diet increased oxidative stress (increased plasma TBARS, reduced SOD activity and increased 3-nitrotyrosine-positive area).

In contrary, Panchal* et al*. [203] demonstrated positive effects of VCO (200 g/kg in diet for 16 weeks) in high-carbohydrate diet-induced metabolic syndrome in male Wistar rats; VCO decreased body weights and blood glucose levels and, importantly, it reduced systolic BP, diastolic stiffness and improved the heart structure and function.

#### 6.3.2 Evidence from Clinical Studies

The only one clinical study by Vijayakumar* et al*. [215] examined the effect of coconut oil on heart health in humans which compared the effect of coconut versus sunflower oil (15% of daily calories used as cooking media for 2 years) on cardiovascular risk factors in patients with stable coronary heart disease. The data showed no significant changes in any parameters measured during the whole study: no differences in the anthropometric (body weight, BMI, waist/hip ratio, % of body fat) and biochemical parameters (lipid profile, carrier proteins), vascular function (flow-mediated vasodilatation), antioxidant and anti-inflammatory markers, incidence of cardiovascular events (death, MI, stroke, repeat revascularization) pointing to no beneficial effects of coconut oil on heart health when compared to sunflower oil.

## 7. The Effect of Other Polyphenol-Rich Oils on Cardiovascular Health and Diseases

In addition to major polyphenol-rich oils, a few studies demonstrated positive effects of less frequently examined oils on the cardiovascular system. For example, it has been demonstrated that argan oil reduced BP [216], improved oxidative status, reduced body weight and decreased levels of plasma TG and blood lipoproteins (total cholesterol, LDL — cholesterol) in rats [217, 218] and also in humans [219, 220, 221, 222, 223]. Further, avocado oil was shown to reduce levels of plasma TG, TC, VLDL, LDL, CRP levels [224, 225] and can also prevent the production of ROS in diabetic rats [226, 227]. Positive effects of another polyphenol-rich oil, garlic oil, were demonstrated by increasing cardiac antioxidant enzyme activity, reducing TBARS, decreasing serum cardiac damage markers enzymes (such as LDH, CK-MB, and cTnC) and inflammatory markers in rats (IL-1β, TNF-α, IL-6, NF-κB p65) [228]. In addition, garlic oil was also proved to attenuate hypercholesterolemia, decrease tissue cholesterol, and to reduce atheromatous changes in the aorta [229] and cardiac apoptosis [230] in hypercholesterolemic rats, thus protecting heart from diabetic cardiomyopathy [231]. Supplementation with grape seed oil exerted anti-inflammatory and cardioprotective effect against ISO-induced ischemia [232]. Evening primrose oil improved cardiac recovery after MI in hypercholesterolemic rats and ameliorated platelet aggregation [233, 234] and reduced the systolic BP, serum TG and total cholesterol [235]. Finally, sea buckthorn seed oil showed anti-atherogenic properties in normal and hypercholesterolemic rabbits [236] and protective effect against myocardial ischemia-reperfusion injury in rats through Akt/eNOS signaling pathway [237] as well as exerted an anti-aggregation effect in normolipidemic patients [238].

## 8. Conclusions

There is extending evidence that the type of food oil preferred in the diet (either freshly consumed or used for cooking) may significantly influence human health including the occurrence of cardiovascular and cardiometabolic diseases. In addition to different composition of FA in particular oils, the content of phenolic compounds with known beneficial properties including antioxidant, anti-inflammatory or anti-coagulant, may significantly contribute to their health beneficial effects including effects on cardiovascular system. The most polyphenol-enriched food oils are olive, flaxseed, sesame, soybean and coconut oils with their main phenolic compounds oleuropein, hydroxytyrosol, SDG, sesamin, sesamol and various phenolic acids.

The review of literature documenting cardiovascular effects of above mentioned polyphenol-enriched food oils, as well as couple of additional minor oils with phenolic content, revealed that the effects of particular oils may significantly differ, since both positive and neutral, and even negative effects of these oils on cardiovascular and cardiometabolic diseases were documented. In particular, olive and sesame oils seem to exert anti-hypertensive, anti-atherogenic and cardio- and vasculo-protective effects which are suggested to be at least in part due to their polyphenol content. Flaxseed oil also exerts anti-hypertensive, anti-atherogenic and cardioprotective effects; however, the role of its main polyphenol SDG in its cardiovascular effects is controversial. The effects of coconut and soybean oils on cardiovascular health are ambiguous. Coconut oil has been shown to exert anti-hypertensive effect that seems to be at least partially attributed to its polyphenol contend but its cardiac effects are inconclusive since both positive and negative effects on myocardial structure and function have been documented. Soybean oil seems to be the most controversial among the reviewed oils regarding its effects on cardiovascular health since both anti-atherogenic and pro-atherogenic effects of this oil were documented. Moreover, in some studies where soybean oil served as control oil to other lipidic food components, soybean oil was found less beneficial for cardiovascular health than examined lipidic compounds (e.g., CLA-enriched ghee or Omegaven (a fish oil-based emulsion)).

Taken together, polyphenol-rich food oils seem to represent a non-homogenous group with diverse effects on cardiovascular and cardiometabolic health that are mainly positive, but might be also neutral, and even negative (Table 1, Ref. [16, 17, 18, 21, 22, 30, 31, 32, 33, 34, 36, 37, 38, 39, 40, 43, 45, 47, 48, 49, 52, 55, 56, 59, 65, 66, 69, 70, 71, 72, 73, 77, 78, 80, 81, 82, 83, 84, 89, 90, 91, 93, 94, 95, 110, 111, 112, 113, 114, 120, 121, 122, 123, 124, 148, 159, 160, 162, 166, 167, 169, 179, 189, 201, 203, 206, 209, 210, 211]). Some of the beneficial effects of these oils in cardiovascular system have been documented to be, at least in part, attributed to their polyphenol content but it couldn’t be concluded that phenolic compounds are the major or the only one components responsible for positive cardiovascular effects of food oils.

**Table 1.** **Outline of the cardiovascular effects of major polyphenol-rich food oils**.

Oil	Main phenolic components	Experimental model	Cardiovascular effects	References
Olive oil	Oleuropein	Human	Cardioprotective in I/R	[16, 17, 18]
	Hydroxytyrosol		Anti-hypertensive	[30, 31, 32, 33, 34, 36, 37]
	Tyrosol		Anti-atherogenic	[16, 47, 48, 49, 52]
		Rat	Cardioprotective in I/R	[21, 22]
			Vasculo-protective	[39, 40]
			Anti-atherogenic	[38, 39, 55]
			Anti-hypertensive	[43, 45]
		Mouse	Anti-atherogenic	[59]
		Rabbit	Anti-atherogenic	[56]
Flaxseed oil	Secoisolariciresinol diglucoside (SDG)	Human	Anti-hypertensive	[77, 78, 80]
	Matairesinol		Anti-atherogenic	[89, 90, 91]
	Lariciresinol	Rat	Anti-hypertensive	[65, 66, 69, 70, 71, 72, 73]
	Pinoresinol		Anti-atherogenic	[69, 81]
			Cardioprotective in I/R	[69, 93, 94, 95]
		Mouse	Anti-atherogenic	[82, 83, 84]
Soybean oil	P-hydroxybenzoic acid	Hamster	Anti-atherogenic	[111]
	Vanillic acid	Mouse	Anti-atherogenic	[112]
	Caffeic acid		Pro-atherogenic	[114]
	P-coumaric acid	Rat	Anti-hypertensive	[123, 124]
	Ferulic acid		Anti-atherogenic	[110, 123]
	Sinapic acid		Pro-atherogenic	[113]
		Human	Anti-atherogenic	[120, 121, 122]
Sesame oil	Sesamin,	Rat	Anti-hypertensive	[148]
	Episesamin			
	Sesamol		Cardioprotective in I/R	[179]
	Sesamolin	Human	Anti-hypertensive	[159, 160, 162]
	Sesamolinol	Mouse	Anti-atherogenic	[166, 167, 169]
Coconut oil	Caproic acid	Rat	Anti-hypertensive	[201, 203]
	Caprylic acid			
	Capric acid		Anti-atherogenic	[189, 206, 209, 210]
	Lauric acid	Human	Anti-atherogenic	[211]

I/R, ischemia-reperfusion.
